# Effect of various staining beverages on the color stability of CAD/CAM PMMA denture teeth: An in vitro study

**DOI:** 10.1002/cre2.869

**Published:** 2024-03-03

**Authors:** Ehab Alouch, Mawia Karkoutly, Omar Teriaky

**Affiliations:** ^1^ Department of Removable Prosthodontics Damascus University Damascus Syrian Arab Republic; ^2^ Department of Pediatric Dentistry Damascus University Damascus Syrian Arab Republic

**Keywords:** coffee, computer‐aided design/computer‐aided manufacturing, esthetics, tea

## Abstract

**Objective:**

This study aimed to compare the color change of computer‐aided design (CAD)/computer‐aided manufacturing (CAM) polymethyl methacrylate (PMMA) denture teeth and conventional acrylic teeth after immersion in three staining beverages (coffee, red tea, and cola) for a day, 7 days, and 30 days.

**Materials and Methods:**

Group 1: Conventional acrylic teeth (*n* = 32). Group 2: Milled CAD/CAM teeth out of PMMA disc (*n* = 32). The specimens of each material were further divided into four subgroups: (1) Control group, distilled water (*n* = 16). (2) Red tea solution (*n* = 16). (3) Coffee solution (*n* = 16). (4) Cola (*n* = 16). The color change (∆E) was assessed using a spectrophotometer at four time points: at the baseline (t_0_), on the 1st day (t_1_), on the 7th day (t_2_), and the 30th day (t_3_) of immersion. Kolmogorov–Smirnov test was applied, followed by performing independent samples *t* test, one‐way analysis of variance and post‐hoc Tukey tests to compare the color change values at different time points.

**Results:**

The mean score of NBS values of the coffee solution indicates perceivable color change at the end of the 30th day in the conventional acrylic teeth group. It was 0.843 ± 0.395 at t_1_, then increased to 1.017 ± 0.477 at t_2_ and to 2.259 ± 1.059 at t_3_. There is a statistically significant difference (*p* < 0.05) in color change values between both tooth types at the end of the 30th day of immersion in red tea solution and a statistically significant difference at the end of the 7th day (*p* < 0.05) and the 30th day (*p* < 0.05) of immersion in coffee solution.

**Conclusions:**

CAD/CAM PMMA teeth are more color stable than conventional acrylic teeth after 30 days of immersion in coffee and red tea solution.

## 1 INTRODUCTION

Although the proportion of complete edentulous patients has decreased, the percentage of partial edentulism is increasing due to the increased life expectancy (Al‐Rafee, 2020). According to the World Health Organization, the number of people aged 60 years or over has been increasing rapidly in recent years (Al‐Rafee, 2020; Rudnicka et al., 2020). Fixed dental prostheses (FDPs) and implant‐supported fixed prostheses are the most appropriate treatment options for partially edentulous patients. However, FDPs are contraindicated when there are unfavorable supporting abutments and multiple missing teeth (Alenezi & Aloqayli, 2023). In addition, dental implants are a high‐cost and time‐consuming option, especially when the implant site requires additional therapies (Alenezi & Aloqayli, 2023). In contrast, removable partial dentures (RPDs) are time and cost savings, provide aesthetic support, and are viable rehabilitation options for long edentulous spans. Therefore, the use of RPDs is still necessary, and it will be growing even more in the future (Mousa et al., 2021).

Conventional acrylic resin artificial teeth are widely used in prosthetic rehabilitation due to their advantages, such as biocompatibility, high flexural strength, fracture resistance, and bond strength to acrylic resin denture bases. In addition, they have good elastic properties due to their ability to absorb masticatory forces. However, they present aesthetic problems since they have low abrasion wear resistance and high staining susceptibility (Muhammad et al., 2022). Polymethyl methacrylate (PMMA) is a synthetic polymer free of inorganic fillers, which was introduced in 1936, and it is widely used for various dental applications owing to its unique properties. PMMA has several advantages, such as good physical and mechanical properties, low density, low cost, and biocompatibility. However, adding fibers or nanoparticles can compromise the aesthetic properties of PMMA, such as color and translucency. In addition, PMMA presents inferior wear resistance, which leads to staining after some time (Zafar, 2020). Computer‐aided design (CAD)/computer‐aided manufacturing (CAM) technology reduces chairside time, improves the aesthetic and mechanical properties of dental materials, and enhances the quality of different prosthetic restorations. However, the CAD/CAM technique is highly expensive and requires trained staff (Alghazzawi, 2016; Islam et al., 2023). CAD/CAM PMMA is superior to conventionally heat‐cured PMMA in terms of mechanical features such as impact strength, flexural strength, hardness, and flexural modulus. Regarding biocompatibility, CAD/CAM PMMA is biocompatible with a minimal leaching residual monomer. In addition, CAD/CAM PMMA was superior to conventional heat‐cured PMMA surface hardness, surface wettability, surface roughness, and hydrophobicity, which affect acrylic discoloration (Al‐Dwairi et al., 2020; Zafar, 2020). Therefore, this study aimed to evaluate and compare the color change of CAD/CAM PMMA denture teeth and conventional acrylic teeth after immersion in three staining beverages (coffee, red tea, and cola) for a day, 7 days, and 30 days. The null hypothesis was that no statistically significant difference would be detected in the color stability between conventional heat‐cured PMMA and CAD/CAM PMMA after immersion in various staining beverages at different times.

## 2 MATERIALS AND METHODS

### 2.1 Sample size and grouping

This was an in‐vitro study. The sample size was calculated using the G.Power 3.1.9 software (G*Power 3.1.9, Heinrich Hein Universität Düsseldorf, Düsseldorf, Germany). Effect size *f* = 0.50/*α* err prob = 0.05/Power (1 − *β* err prob) = 0.80/Number of groups = 8. A sample size of 64 samples was obtained. A sample size of 64 specimens achieved an effect size *f* of (0.50), 80% Power (1 ‐ *β* err prob), and a significance level of 0.05. A pilot study on eight specimens was conducted to determine the effect size. The samples corresponded to maxillary central and lateral incisors. The tooth shade was A1, and the size was S3. Two types of acrylic teeth were used: Group 1: Conventional acrylic teeth (NAPERCE; Yamahachi Dental Mfg. Co.) (*n* = 32). Group 2: Milled CAD/CAM teeth out of PMMA disc (Polywax, Bilkim Ltd. Co.) (*n* = 32). The specimens of each material were further divided into four subgroups according to the immersing solution as follows: (1) Control group, distilled water (Pure Water, Pure Water Co.) (*n* = 16). (2) Red tea (Cherry Brand, Sweid Co.) (*n* = 16). (3) Coffee (Café Shami, Al‐Shami‐Cafe Co.) (*n* = 16). (4) Cola (Pepsi, PepsiCo, Inc.) (*n* = 16). The primary endpoint was the color change of CAD/CAM PMMA denture teeth and conventional acrylic teeth after immersion in the study medium for a day, 7 days, and 30 days.

### 2.2 Immersion solutions and specimen preparation

To prepare the tea solution, two tea bags (2 × 2 g) were immersed in boiling water, and the final volume of the tea solution was 300 mL following the tea waste removal. The coffee solution was prepared by solving 2 g of coffee in 100 mL of boiling water, and both solutions were filtered and left until they cooled to 37°C. Moreover, 100 mL of cola and distilled water were poured as received (Al‐Shami et al., 2023). All solutions were poured into separate dishes, stored in an incubator (Classic Incubators, LEEC Ltd.) at 37°C, and stirred three times a day to avoid sedimentation. Every day, the immersing solutions were replaced to prevent contamination (Al‐Shami et al., 2023).

**Figure 1:**
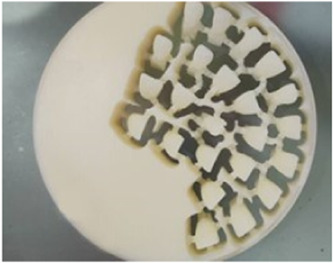
Milled CAD/CAM teeth out of PMMA disc.

The specimens of the second group were milled out of PMMA disc using a CAD/CAM dental milling machine (DWX‐52DCi, Ronald DGA Co.) (Figure 1) (Soeda et al., 2022), and the milled teeth were corresponding in size, shape, and color to the conventional acrylic teeth. The teeth were immersed in the study solution for a day, 7 days, and 30 days (Aydın et al., 2020). Each medium contains eight specimens of each group with no contact with each other (Aydın et al., 2020).

### 2.3 Color assessment

The color change (∆E) was assessed using a spectrophotometer (VITA Easyshade® V, VITA North America) using a white background (Takhtdar et al., 2023). The Kappa coefficient of intraexaminer reliability was >0.8. It was evaluated at four‐time points: at the baseline (t_0_), on the 1st day (t_1_), on the 7th day (t_2_), and on the 30th day (t_3_) of immersion (Aydın et al., 2020), and repeated measurements were standardized in a positioning jig (Positioning Jigs, MISUMI). Before each measurement, a white calibration plate (Labsphere's Spectralon®, Labsphere, Inc.) was used (Takhtdar et al., 2023). ∆E was calculated using the following formula:

$$∆E=\sqrt{\left[{\left(∆L\right)}^{2}+{\left(∆a\right)}^{2}+{\left(∆b\right)}^{2}\right]},$$

where ∆L, ∆a, and ∆b indicate lightness, green/red coordinate, and blue/yellow coordinate, respectively (Al‐Shami et al., 2023). ∆E values were converted to National Bureau of Standards (NBS) units to relate the ∆E data to a clinical environment using the following equation:

NBS units=∆E×0.92.

The critical remarks on color differences of NBS units were as follows:

0.0–0.5 = Trace: extremely slight change.

0.5–1.5 = Slight: slight change.

1.5–3.0 = Noticeable: perceivable.

3.0–6.0 = Appreciable: marked change.

6.0–12.0 = Much: extremely marked change.

12.0 or more = Very much: change to another color (Koksal & Dikbas, 2008).

### 2.4 Statistical analysis

IBM SPSS software version 24 (IBM SPSS Statistics® version 24, IBM Corp.) was used to perform statistical analysis. Kolmogorov–Smirnov test was applied to check the normality of data, followed by performing an independent samples *t* test to compare the color change values of both tooth types in the study medium at different time points. One‐way analysis of variance (ANOVA) and post‐hoc Tukey tests were performed to compare the cumulative effect of the study medium at different time points in each group. Descriptive statistics were presented as mean, standard deviation, standard error, minimum, and maximum. The level of significance was set at 0.05 (*p* < 0.05).

## 3 RESULTS

Descriptive statistics of the color change values of study groups at different time points are listed in Table 1. The mean score of NBS values of the coffee solution indicates perceivable color change at the end of the 30th day (2.259 ± 1.059) in the conventional acrylic teeth group. Regarding red tea and cola, the mean score of NBS values refers to a slight color change for both tooth types at different time points. In the cola immersion group, the mean score of NBS value for CAD/CAM PMMA teeth was 0.655 ± 0.307 at t_1_, then increased to 0.870 ± 0.408 at t_2_ and to 0.936 ± 0.439 at t_3_. Similarly, the mean score of NBS value for conventional acrylic teeth was 0.645 ± 0.302 at t_1_, then increased to 0.870 ± 0.408 at t_2_ and to 0.934 ± 0.438 at t_3_. Therefore, the level of discoloration of CAD/CAM PMMA teeth and conventional acrylic teeth increased with time after immersion in cola beverage. In addition, regarding conventional acrylic teeth, the mean score of NBS value was 0.714 ± 0.335 at t_1_, then increased to 0.952 ± 0.446 at t_2_ and to 1.529 ± 0.717 at t_3_ after immersion in red tea solution. Similarly, the mean score of NBS value was 0.843 ± 0.395 at t_1_, then increased to 1.017 ± 0.477 at t_2_ and to 2.259 ± 1.059 at t_3_ after immersion in coffee solution. Hence, the level of discoloration of conventional acrylic teeth increased with time after immersion in red tea and coffee solution. Regarding the distilled water group, the mean scores of NBS values indicate an extremely slight color change.

**Table 1:** Descriptive statistics of the color change values of both tooth types in the study medium at different time points.

Medium	Time points	Type of tooth	Mean ± SD	SE	Min	Max
Red tea	t_1_	CAD/CAM PMMA teeth	0.717 ± 0.336	0.059	0.056	1.442
		Conventional acrylic teeth	0.714 ± 0.335	0.059	0.056	1.436
	t_2_	CAD/CAM PMMA teeth	0.952 ± 0.446	0.079	0.074	1.914
		Conventional acrylic teeth	0.952 ± 0.446	0.079	0.074	1.914
	t_3_	CAD/CAM PMMA teeth	0.153 ± 0.072	0.013	0.012	0.308
		Conventional acrylic teeth	1.529 ± 0.717	0.127	0.119	3.074
Coffee	t_1_	CAD/CAM PMMA teeth	0.856 ± 0.401	0.071	0.066	1.720
		Conventional acrylic teeth	0.843 ± 0.395	0.070	0.065	1.694
	t_2_	CAD/CAM PMMA teeth	0.105 ± 0.049	0.009	0.008	0.212
		Conventional acrylic teeth	1.017 ± 0.477	0.084	0.079	2.044
	t_3_	CAD/CAM PMMA teeth	0.435 ± 0.204	0.036	0.034	0.874
		Conventional acrylic teeth	2.259 ± 1.059	0.187	0.176	4.542
Cola	t_1_	CAD/CAM PMMA teeth	0.655 ± 0.307	0.054	0.051	1.316
		Conventional acrylic teeth	0.645 ± 0.302	0.053	0.050	1.296
	t_2_	CAD/CAM PMMA teeth	0.870 ± 0.408	0.072	0.068	1.748
		Conventional acrylic teeth	0.870 ± 0.408	0.072	0.068	1.748
	t_3_	CAD/CAM PMMA teeth	0.936 ± 0.439	0.078	0.073	1.882
		Conventional acrylic teeth	0.934 ± 0.438	0.077	0.073	1.878
Distilled water	t_1_	CAD/CAM PMMA teeth	0.041 ± 0.019	0.003	0.003	0.083
		Conventional acrylic teeth	0.037 ± 0.017	0.003	0.003	0.074
	t_2_	CAD/CAM PMMA teeth	0.161 ± 0.076	0.013	0.013	0.324
		Conventional acrylic teeth	0.160 ± 0.075	0.013	0.012	0.322
	t_3_	CAD/CAM PMMA teeth	0.425 ± 0.199	0.035	0.033	0.854
		Conventional acrylic teeth	0.423 ± 0.198	0.035	0.033	0.850

**Figure 2:**
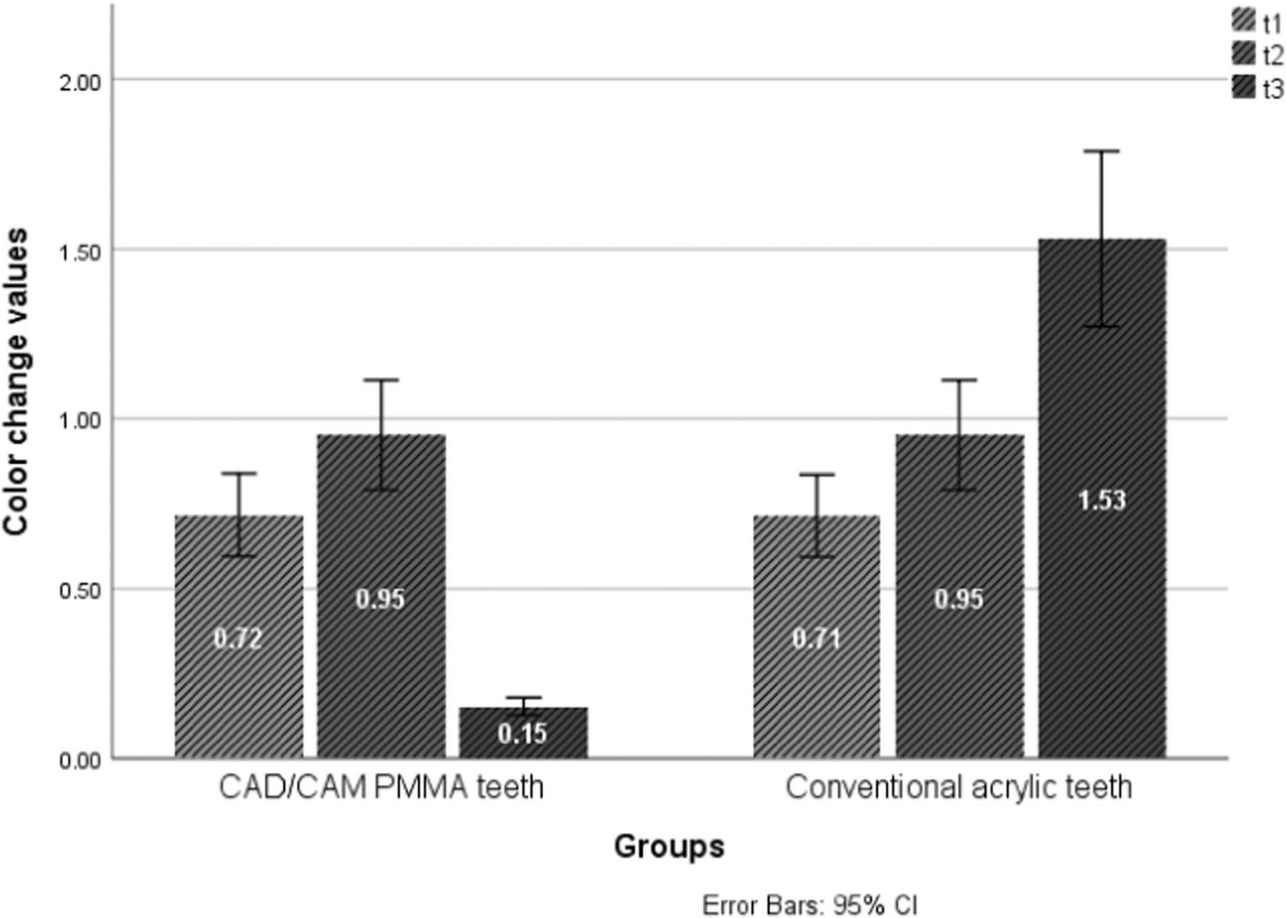
A histogram of color change values of both tooth types in red tea solution at different time points.

**Figure 3:**
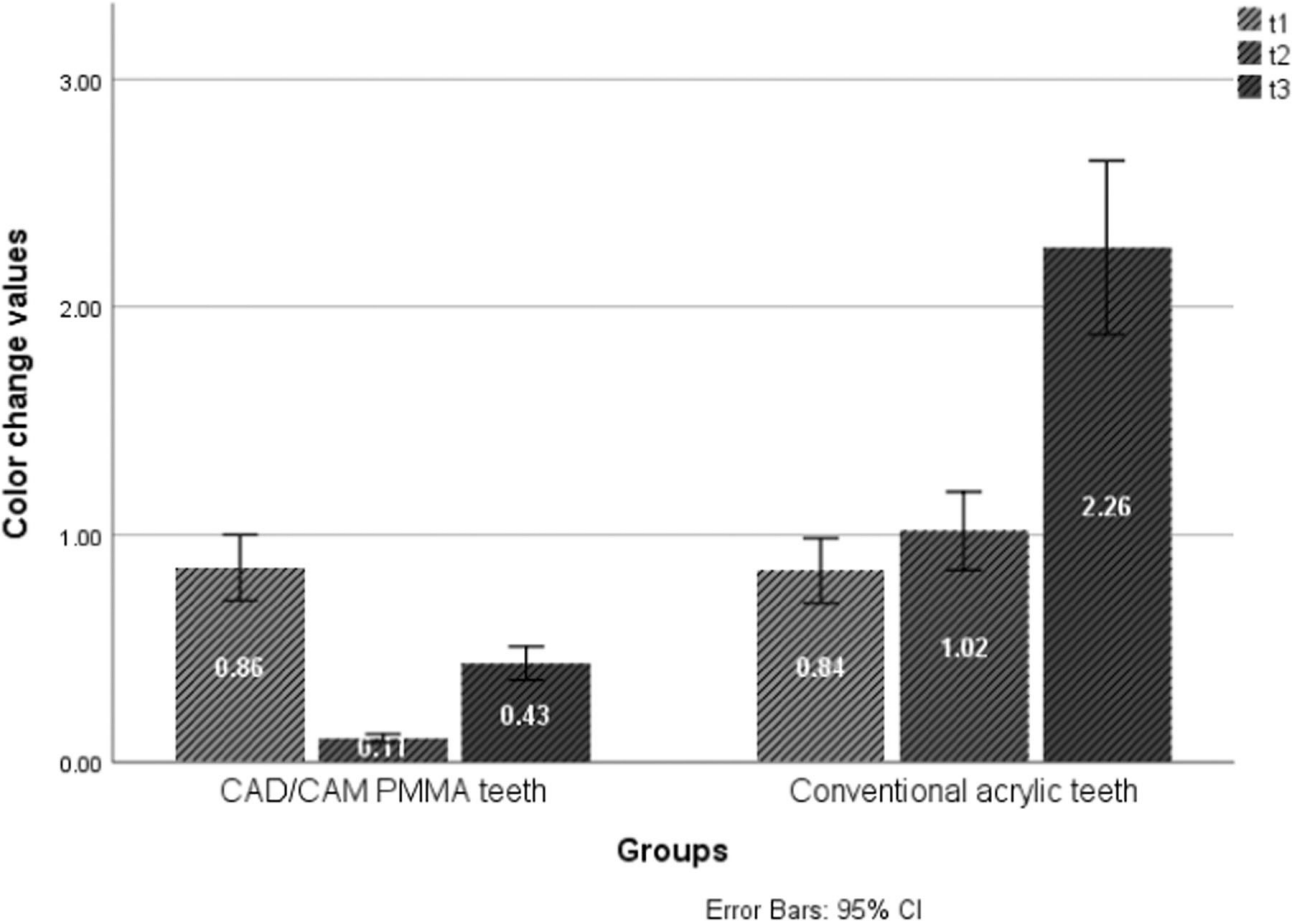
A histogram of color change values of both tooth types in coffee solution at different time points.

The results of the independent samples *t* test showed a statistically significant difference (*p* < 0.05) in color change values between both tooth types at the end of the 30th day of immersion in red tea solution. However, no statistically significant difference was noted at the end of the 1st day (*p* = 0.972) and the 7th day (*p* = 1.000) of immersion (Table 2 and Figure 2). In the coffee solution immersion group, there was a statistically significant difference between the two types of teeth at the end of the 7th day (*p* < 0.05) and the 30th day (*p* < 0.05). However, no statistically significant difference was noted at the end of the 1st day (*p* = 0.897) (Table 3 and Figure 3). No statistically significant difference was noted between the two types of teeth at t_1_ (*p* = 0.896), t_2_ (*p* = 1.000) and t_3_ (*p* = 0.986) in the cola beverage immersion group (Table 4). Similarly, no significant difference was detected at t_1_ (*p* = 0.343), t_2_ (*p* = 0.962), and t_3_ (*p* = 0.971) in the distilled water immersion group (Table 5).

**Table 2:** Results of independent samples *t* test for comparison of color change values of both tooth types in red tea solution at different time points.

Time points	Type of tooth	Mean difference	*t* Value	*p* Value
t_1_	CAD/CAM PMMA teeth	0.003	0.04	0.972
	Conventional acrylic teeth			
t_2_	CAD/CAM PMMA teeth	0.000	0.00	1.000
	Conventional acrylic teeth			
t_3_	CAD/CAM PMMA teeth	−1.376	−10.81	<0.001*
	Conventional acrylic teeth			

* Significant difference at *p* < 0.05.

**Table 3:** Results of independent samples *t* test for comparison of color change values of both tooth types in coffee solution at different time points.

Time points	Type of tooth	Mean difference	*t* Value	*p* Value
t_1_	CAD/CAM PMMA teeth	0.013	0.13	0.897
	Conventional acrylic teeth			
t_2_	CAD/CAM PMMA teeth	−0.912	−10.76	<0.001*
	Conventional acrylic teeth			
t_3_	CAD/CAM PMMA teeth	−1.825	−9.57	<0.001*
	Conventional acrylic teeth			

* Significant difference at *p* < 0.05.

**Table 4:** Results of independent samples *t* test for comparison of color change values of both tooth types in cola beverage at different time points.

Time points	Type of tooth	Mean difference	*t* Value	*p* Value
t_1_	CAD/CAM PMMA teeth	0.010	0.13	0.896
	Conventional acrylic teeth			
t_2_	CAD/CAM PMMA teeth	0.000	0.00	1.000
	Conventional acrylic teeth			
t_3_	CAD/CAM PMMA teeth	0.002	0.02	0.986
	Conventional acrylic teeth			

**Table 5:** Results of independent samples *t* test for comparison of color change values of both tooth types in distilled water at different time points.

Time points	Type of tooth	Mean difference	*t* Value	*p* Value
t_1_	CAD/CAM PMMA teeth	0.004	0.96	0.343
	Conventional acrylic teeth			
t_2_	CAD/CAM PMMA teeth	0.001	0.05	0.962
	Conventional acrylic teeth			
t_3_	CAD/CAM PMMA teeth	0.002	0.04	0.971
	Conventional acrylic teeth			

Results of a one‐way ANOVA post‐hoc Tukey test for comparison of color change values after teeth immersion in the study medium are listed in Tables 6 and 7. Red tea solution had the highest cumulative effect after 30 days of immersion compared with other mediums (*p* < 0.05) in the CAD/CAM PMMA teeth group (Table 6). Regarding the conventional acrylic teeth group, all staining beverages had a cumulative effect after 30 days of immersion compared with distilled water (*p* < 0.05) (Table 7).

**Table 6:** Results of a one‐way ANOVA post‐hoc Tukey test for comparison of the cumulative effect of the study medium at different time points in the CAD/CAM PMMA teeth group.

				95% Confidence interval	
Time points	Multiple comparisons	Mean difference	*p* Value	Lower bound	Upper bound
t_1_	Red tea solution versus Coffee solution	−0.139	0.071	−0.289	0.012
	Red tea solution versus Cola beverage	0.062	0.411	−0.088	0.213
	Red tea solution versus Distilled water	0.676	<0.001*	0.526	0.826
	Coffee solution versus Cola beverage	0.200	0.009*	0.051	0.351
	Coffee solution versus Distilled water	0.814	<0.001*	0.664	0.965
	Cola beverage versus Distilled water	0.613	<0.001*	0.463	0.764
t_2_	Red tea solution versus Coffee solution	0.846	<0.001*	0.696	0.998
	Red tea solution versus Cola beverage	0.082	0.282	−0.069	0.234
	Red tea solution versus Distilled water	0.791	<0.001*	0.640	0.943
	Coffee solution versus Cola beverage	−0.764	<0.001*	−0.915	−0.613
	Coffee solution versus Distilled water	−0.055	0.468	−0.207	0.096
	Cola beverage versus Distilled water	0.708	<0.001*	0.557	0.860
t_3_	Red tea solution versus Coffee solution	−0.281	<0.001*	−0.412	−0.151
	Red tea solution versus Cola beverage	−0.782	<0.001*	−0.914	−0.652
	Red tea solution versus Distilled water	−0.271	<0.001*	−0.402	−0.141
	Coffee solution versus Cola beverage	−0.501	<0.001*	−0.632	−0.370
	Coffee solution versus Distilled water	0.010	0.878	−0.121	0.141
	Cola beverage versus Distilled water	0.511	<0.001*	0.381	0.642

* Significant difference at *p* < 0.05.

**Table 7:** Results of a one‐way ANOVA post‐hoc Tukey test for comparison of the cumulative effect of the study medium at different time points in the conventional acrylic teeth group.

				95% Confidence interval	
Time points	Multiple comparisons	Mean difference	*p* Value	Lower bound	Upper bound
t_1_	Red tea solution versus Coffee solution	−0.128	0.089	−0.277	0.020
	Red tea solution versus Cola beverage	0.069	0.355	−0.079	0.218
	Red tea solution versus Distilled water	0.677	<0.001*	0.530	0.826
	Coffee solution versus Cola beverage	0.198	0.009*	0.050	0.346
	Coffee solution versus Distilled water	0.805	<0.001*	0.658	0.954
	Cola beverage versus Distilled water	0.607	<0.001*	0.460	0.756
t_2_	Red tea solution versus Coffee solution	−0.064	0.504	−0.256	0.126
	Red tea solution versus Cola beverage	0.082	0.395	−0.109	0.274
	Red tea solution versus Distilled water	0.791	<0.001*	0.601	0.983
	Coffee solution versus Cola beverage	0.147	0.130	−0.044	0.339
	Coffee solution versus Distilled water	0.856	<0.001*	0.665	1.048
	Cola beverage versus Distilled water	0.709	<0.001*	0.518	0.901
t_3_	Red tea solution versus Coffee solution	−0.730	<0.001*	−1.068	−0.392
	Red tea solution versus Cola beverage	0.594	0.001*	0.260	0.933
	Red tea solution versus Distilled water	1.106	<0.001*	0.768	1.444
	Coffee solution versus Cola beverage	1.325	<0.001*	0.987	1.663
	Coffee solution versus Distilled water	1.836	<0.001*	1.499	2.175
	Cola beverage versus Distilled water	0.511	0.003*	0.173	0.850

* Significant difference at *p* < 0.05.

## 4 DISCUSSION

Acrylic removable appliances are still the best treatment option for complete or partial edentulous patients. Therefore, the materials of these dental appliances must have high mechanical and physical properties (Mousa et al., 2021). As the patient's interest in cosmetic aspects has increased, the aesthetic features became an essential requirement for successful prosthetic treatment. Color stability of removable appliances is an important clinical aspect, as the appearance and color of teeth must match the surrounding dental tissue and adjacent teeth, and this is mainly related to the selection of biomaterials that have long‐term color stability. Therefore, the color stability of acrylic teeth and their resistance to staining have become a critical demand (Donovan et al., 2001). With developments in dental materials, including acrylic, many techniques and types of acrylic teeth have emerged, which have increased in popularity due to the noticeable improvement in their properties. All these factors led to the introduction of new types of artificial teeth to suit these new requirements, such as milled CAD/CAM teeth out of PMMA discs (Al‐Dwairi et al., 2020; Zafar, 2020). Hence, the need arose to evaluate the resistance of these teeth to staining when exposed to various beverages. Therefore, this study aimed to compare the color change of CAD/CAM PMMA denture teeth and conventional acrylic teeth after immersion in three staining beverages (coffee, red tea, and cola) for a day, 7 days, and 30 days. To the best of the authors' knowledge, no study has ever evaluated the discoloration of CAD/CAM PMMA teeth compared with conventional acrylic teeth.

In this study, color measurement was performed according to the CIEL*a*b* (CIELAB) color scale using a spectrophotometer. According to Paul et al. (2002)., spectrophotometric shade assessment is more reproducible and accurate than human shade analysis. Coffee, red tea, and cola were assessed in the current study as they are the most consumed staining beverages in the daily diet (Al‐Shami et al., 2023; Aydın et al., 2020), and distilled water was selected as the control group. According to Al‐Qarni et al. (2020) and Dayan et al. (2019), distilled water is a preferred control group when comparing staining beverages. The samples were immersed in the medium for a day, 7 days, and 30 days. The maximum duration of immersion was 30 days, according to many studies (Al‐Shami et al., 2023; Takhtdar et al., 2023) which considered an optimal period to obtain a cumulative effect. In addition, according to Aydin et al. (2020), 30 days of exposure to certain beverages would reproduce 2.5 years of regular consumption, and a day of exposure would reproduce 30 days of consumption.

The result of the current study showed that the mean score of NBS values of the coffee solution indicates perceivable color change at the end of the 30th day in the conventional acrylic teeth group. This result could be attributed to the fact that discoloration results from the presence of yellow dyes and acids, including tannic acid. In addition, acidification of coffee produces high‐molecular‐weight, brown‐colored nitrogenous compounds called melanoidins, which are primarily responsible for discoloration (Nunes & Coimbra, 2007). This finding is in agreement with the one reported by Mutlu‐Sagesen et al. (2001), which suggested that the most staining beverage for porcelain, conventional, and reinforced acrylic teeth is filtered coffee after 30 days of immersion. Likewise, according to Gregorius et al. (2012), coffee beverage causes the largest color shift in hue, value, and chroma for high‐strength acrylic denture teeth after 7 days of immersion.

No statistically significant difference was noted between the two types of teeth at different time points in the cola beverage immersion group, and the mean score of NBS values refers to a slight color change. This result is consistent with Mousavi et al. (2016) finding, which suggested that cola causes a slight color change in three different brands of acrylic teeth after 6 weeks of immersion. In addition, Koksal and Dikbas (2008) concluded that according to NBS values, cola causes a slight color change in acrylic and porcelain denture teeth after 30 days of immersion. Furthermore, Aydin et al. (2020) study found that cola causes staining below the clinically perceptible level in different CAD/CAM resin blocks at the end of the 30th day.

Regarding the coffee and red tea immersion group, the level of discoloration of conventional acrylic teeth increased with time after immersion in coffee and red tea solution compared with CAD/CAM PMMA teeth. The higher staining in conventional acrylic teeth is due to the difference in the structure of each type of tooth, as the molecular structure of acrylic teeth tends to absorb water, which causes pigmentation. Absorbed water molecules separate the polymer chains, which leads to the penetration of the staining beverage (Neppelenbroek et al., 2015). In addition, according to Arslan et al. (2018), conventional heat‐polymerized PMMA exhibits higher hydrophilic properties compared with CAD/CAM PMMA‐based polymers. Polymerization occurs under high pressure and temperature, which reduces the number of residual monomers, thus producing materials with better mechanical properties, fewer pores, and greater color stability (Al‐Dwairi et al., 2020; Zafar, 2020). However, this result is in contrast with Al‐Qarni et al. (2020) finding, which suggested that CAD/CAM PMMA teeth have a similar color change to conventional acrylic teeth when immersed in different staining beverages. The present report evaluated staining and color stability. As previously conducted for other dental materials, future studies are needed to test other important characteristics such as hardness (Colombo et al., 2020), fatigue (Rodríguez‐Ivich et al., 2022), and flexural strength (Cacciafesta et al., 2008) to complete the knowledge about CAD/CAM PMMA denture teeth.

This study has some limitations. The use of specific types of beverages and continuous immersion does not match the oral environment, as laboratory study conditions cannot accurately simulate the oral environment. The coloration is affected by chemical and mechanical changes in the oral cavity, such as the composition of saliva, differences in foods and drinks, mastication efforts, and temperature fluctuations. In addition, abrasion and corrosion can increase discoloration, which also relates to oral health, dietary habits, and the care of the removable denture.

## 5 CONCLUSIONS

Based on our findings, CAD/CAM PMMA teeth are more color stable than conventional acrylic teeth after 30 days of immersion in coffee and red tea solution. However, regarding the cola immersion group, the mean score of NBS values refers to a slight color change for both tooth types at different time points.

## AUTHOR CONTRIBUTIONS

Ehab Alouch collected data, extracted the data, and performed the statistical analysis. Mawia Karkoutly wrote the manuscript and performed the statistical analysis. Omar Teriaky research concept and design, performed critical revision of the manuscript. All authors have read and approved the manuscript.

## CONFLICT OF INTEREST STATEMENT

The authors declare no conflict of interest.

## ETHICS STATEMENT

Ethical approval was provided by the ethics board at Damascus University (N2497).

## Data Availability

The data that support the findings of this study are available from the corresponding author upon reasonable request.
